# Soil Viruses: A New Hope

**DOI:** 10.1128/mSystems.00120-19

**Published:** 2019-05-28

**Authors:** Joanne B. Emerson

**Affiliations:** a Department of Plant Pathology, University of California, Davis, Davis, California, USA

**Keywords:** metagenomics, microbial ecology, soil microbiology, virus

## Abstract

As abundant members of microbial communities, viruses impact microbial mortality, carbon and nutrient cycling, and food web dynamics. Although most of our information about viral communities comes from marine systems, evidence is mounting to suggest that viruses are similarly important in soil. Here I outline soil viral metagenomic approaches and the current state of soil viral ecology as a field, and then I highlight existing knowledge gaps that we can begin to fill. We are poised to elucidate soil viral contributions to terrestrial ecosystem processes, considering: the full suite of potential hosts across trophic scales, the ecological impacts of different viral replication strategies, links to economically relevant outcomes like crop productivity, and measurable *in situ* virus-host population dynamics across spatiotemporal scales and environmental conditions. Soon, we will learn how soil viruses contribute to food webs linked to organic matter decomposition, carbon and nutrient cycling, greenhouse gas emissions, and agricultural productivity.

## Abstract (precis)

As abundant members of microbial communities, viruses impact microbial mortality, carbon and nutrient cycling, and food web dynamics. Although most of our information about viral communities comes from marine systems, evidence is mounting to suggest that viruses are similarly important in soil.

## PERSPECTIVE

Soil and rhizosphere microorganisms play key roles in carbon and nutrient cycling, plant health, and sustainable agriculture (1–3), and we are ready to learn how viruses enhance or inhibit microbial contributions to these processes. Soil viruses are abundant (∼10^7^ to 10^9^ viruses per gram [4]), and we already have evidence for direct and indirect viral impacts on soil microbiota and biogeochemistry (4–8). In marine systems, where viral ecological investigations have been honed for nearly 2 decades, viruses impact global ocean food webs, carbon cycling, and climate (9). Early evidence suggests that viruses play similarly important roles in terrestrial ecosystems, but large-scale soil viromic efforts have only recently become possible (7, 8, 10). We can now assess soil viral diversity at scales of hundreds to thousands of viral populations per sample (7, 10), and we are already approaching diversity saturation for at least the most abundant viruses in the soils studied thus far (7). We have a variety of tools for genome-enabled interrogations of viral ecology across natural and agricultural soils, and we are poised to quantitatively investigate viral impacts on soil ecosystem processes and plant productivity. In this Perspective, I briefly review the state of the art in soil viral ecology and then present a series of fundamental and applied knowledge gaps that we are ready to begin to fill, using soil viral ecological approaches.

A metagenomic and/or metatranscriptomic approach is required for comprehensive viral community ecology in any ecosystem because there is no universal marker gene for viruses. Viral metagenomic approaches have been refined in aqueous systems, where size fractionation through filtration allows for relatively easy recovery of purified viral particles for DNA extraction and sequencing (9). Soil viral ecology has lagged behind these efforts, largely due to the challenge of recovering enough viral DNA (without biased amplification approaches [4]) to facilitate computational metagenomic assembly, which is required for quantitative viral community ecology (4, 7, 9). Although many of the physicochemical differences between oceans and soil are obvious, the links between these differences and the pace of methodological advances are worth describing in more detail.

For example, early marine viromics studies reported filtration of ∼200 liters of seawater per sample in order to recover enough viral DNA for metagenomic sequencing. Processing the equivalent amount of soil would be impractical and expensive, not to mention destructive to field sites (think Swiss cheese) in ways that do not apply to marine systems. In order to facilitate filtration for viral purification from soil, substantial volumes of buffer must be added (an approximately 3:1 buffer/soil ratio is typical [7, 8]). Although buffer chemistry and physical methods for separating viral particles from the soil matrix and other biota have improved (4, 7, 8), it is still not practical to work with more than ∼50 g of soil per sample. Recent bioinformatic advances allow for mining viral sequences from complex microbial genomic and metagenomic data sets, circumventing the need for viral purification in the laboratory (7, 10). However, ongoing work in my group suggests that viral diversity recovered from purified soil viral metagenomes (viromes) is much higher than viral diversity recovered from bulk soil metagenomes. So, although all of these viral ecology-specific advances have certainly helped, library construction from ever-lower DNA inputs is probably the primary facilitator of quantitative soil viromics. It is now possible to extract sufficient viral DNA (∼30 to 100 ng) from only ∼5 to 50 g of (surface) soil for Illumina metagenomic sequencing. Although this amount of soil still does not approach the tiny spatial scales that are relevant to most viral and microbial processes (2), it is a workable amount, and we seem to recover similar viral communities from similar soils and treatments (7, 8).

**FIG 1:**
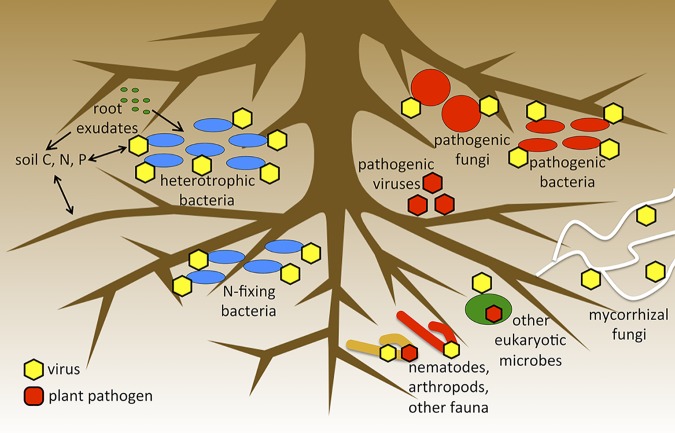
Examples of predicted virus-host interactions, potential feedback between viruses and biogeochemistry, and possible viral impacts on plant pathogens that are ripe for further investigation in a variety of natural and agricultural soils; not to scale; food web dynamics and nutrient cycling are not accurately depicted across trophic scales.

While we assume that viruses of bacteria (bacteriophages) dominate soil viral communities (4, 11), our methods are inherently biased against the recovery of mycoviruses, plant viruses, viruses of (some) plant pathogens and their vectors, and viruses of other soil fauna. All of these viruses are presumably present in at least some soils and have the potential for substantial, as-yet-unknown ecological impacts (Fig. 1). The vast majority of known plant and fungal viruses have RNA genomes, which by nature cannot be detected in viral size-fraction DNA metagenomes, so we are developing laboratory methods for recovering RNA viral sequences from soil. Computationally, some of the most useful pipelines for viral ecology, including tools for the identification of viral sequences in genomic and metagenomic data sets and for viral taxonomic assignments, are designed for viruses of bacteria and archaea. While software is also available for the recovery of eukaryotic viruses from metagenomic data, our ability to recognize eukaryotic viral sequences that diverge from those in public databases is limited (11), as is our ability to bioinformatically predict a specific eukaryotic host for such viruses. Populating public databases with more viral genomic sequences across host trophic scales is essential to our ability to study soil viral ecology holistically. This can be accomplished through cultivation combined with omics, by mining existing organismal DNA and RNA sequencing data for viral sequences, and by strategic sequencing of new and existing sample collections for this purpose.

Viruses of bacteria and archaea typically undergo one of two replication cycles (the lytic or the lysogenic cycle [12]), and we are ready to understand the ecological ramifications of each, along with conditions that drive shifts between the two in soil. In the lytic cycle, viral infection leads to near-immediate viral replication inside the host cell and results in cell lysis upon the release of progeny viruses. In the lysogenic cycle, viral DNA is inserted into the host chromosome or maintained extrachromosomally and replicated passively with the host, unless/until the virus is induced to undergo the lytic cycle. These replication strategies likely have important implications for biogeochemistry, microbial evolution, and microbial community composition and function. For example, lysis results in host mortality, impacting metabolic functions performed by lysed host populations, and it contributes to nutrient cycling by releasing host cellular contents into the environment. Viral “auxiliary metabolic genes” (e.g., those involved in carbon cycling [7, 9]) can be expressed during the infection cycle with direct impacts on biogeochemistry, and both replication strategies can facilitate horizontal gene transfer. It has been hypothesized that temperate viruses (those capable of lysogeny) dominate in soil environments (6), and my group is working to test this hypothesis. If temperate viruses are abundant in soil, we want to know under what conditions they are induced and whether regular shifts from lysogeny to lysis might make soil viral ecology fundamentally different from lysis-dominated marine viral ecology. Devoting further attention to the ecological consequences of other forms of viral replication, such as inefficient lytic infections on alternative hosts, is also warranted, as discussed elsewhere (12).

Soil and rhizosphere viral contributions to agricultural ecosystem function and economically important agricultural outcomes, like crop health, yield, and quality, have not been explored in detail. Finding appropriate and feasible measurements to link soil viruses to crop yields at relevant scales is a difficult problem that applies to other microorganisms, too. Long-term agricultural research sites, such as the Russell Ranch Sustainable Agriculture Research Facility at UC Davis (https://asi.ucdavis.edu/programs/rr), can offer integrated instrumentation, sensors, and remote sensing data to precisely track inputs (e.g., water, fertilizer, soil parameters, and meteorological conditions) linked to crop productivity and quality. Interestingly, such data sets have demonstrated unknown bottlenecks that restrict microbial nitrogen and phosphorus cycling to bioavailable forms for crop plants, particularly for modern high-productivity maize varieties (13). Low nutrient turnover and inadequate release of nutrients from organic pools have become particular problems in cover-cropped systems, in which soil nitrogen tends to be immobilized (14), and viral lysis could conceivably play a key role in the liberation of nutrients tied up in microbial biomass. Piggybacking on existing large-scale agricultural studies may be an effective path toward integrating soil viral and microbial ecological analyses to identify soil management practices that enhance nutrient bioavailability to crop plants.

In our recent collaborative study of viral ecology in thawing permafrost soils, we demonstrated that virus-host abundance patterns can be tracked in metagenomic data (7). We used sequence homology-based methods (e.g., CRISPRs) for virus-host linkages that do not rely on known taxonomy of the virus or the host (7, 10). Our results suggested that patterns of soil virus-host dynamics can differ by microbial host lineage. For some host lineages, viruses appeared to be more successful predators in a more thawed habitat, while viruses that infected other host lineages seemed more successful in a less thawed habitat. It is too early to make sweeping generalizations about virus-host dynamics beyond these specific examples from a single ecosystem, but it is exciting that we now have the means to link reconstructed viral population genomes to metagenome-assembled genomes (MAGs) from their microbial hosts. These virus-host linkages (identified by bioinformatics alone) are ripe for further interrogation and confirmation (15), e.g., through cultivation, microfluidics, and the identification of proviruses in long-read microbial metagenomic sequencing data. Also, the extent to which viral abundances in metagenomes reflect activity and infectivity remains to be seen (16). Still, these early results open the door to taxonomically and metabolically resolved investigations of how viral infection events fit into the larger framework of trophic cascades and ecosystem function in soil.

In the next 5 years, I expect that a more comprehensive understanding of viral diversity, ecology, and activity across a range of natural and agricultural soils will bring us closer to our ultimate goals of predicting and manipulating viral impacts on microbial ecology, carbon and nutrient cycling, and plant productivity in terrestrial ecosystems.
